# No Evidence for the Association between a Polymorphism in the *PCLO* Depression Candidate Gene with Memory Bias in Remitted Depressed Patients and Healthy Individuals

**DOI:** 10.1371/journal.pone.0112153

**Published:** 2014-11-07

**Authors:** Janna N. Vrijsen, Anne Speckens, Alejandro Arias-Vásquez, Barbara Franke, Eni S. Becker, Iris van Oostrom

**Affiliations:** 1 Department of Psychiatry, Radboud University Medical Centre, Nijmegen, the Netherlands; 2 Donders Institute for Brain, Cognition and Behaviour, Radboud University Medical Centre, Nijmegen, the Netherlands; 3 Department of Cognitive Neuroscience, Radboud University Medical Centre, Nijmegen, The Netherlands; 4 Department of Human Genetics, Radboud University Medical Centre, Nijmegen, The Netherlands; 5 Behavioural Science Institute, Radboud University Nijmegen, Nijmegen, The Netherlands; Max Planck Institute of Psychiatry, Germany

## Abstract

The *PCLO* rs2522833 candidate polymorphism for depression has been associated to monoaminergic neurotransmission. In healthy and currently depressed individuals, the polymorphism has been found to affect activation of brain areas during memory processing, but no direct association of *PCLO* with memory bias was found. We hypothesized that the absence of this association might have been obscured by current depressive symptoms or genetically driven individual differences in reactivity to stressful events. Experiencing stressful childhood events fosters dysfunctional assumptions that are related to cognitive biases, and may modulate the predisposition for depression via epigenetic effects. The association between *PCLO* and memory bias, as well as interaction between *PCLO* and childhood events was studied in patients remitted from depression (N = 299), as well as a sample of healthy individuals (N = 157). The participants performed an emotional verbal memory task after a sad mood induction. Childhood trauma and adversity were measured with a questionnaire. The Genotype main effect, and Genotype by Childhood Events interaction were analyzed for memory bias in both samples. *PCLO* risk allele carrying remitted depressed patients did not show more negatively biased memory than non-risk allele carriers, not even patients with stressful childhood events. A similar pattern of results was found in healthy individuals. Memory bias may not be strongly associated with the *PCLO* rs2522833 polymorphism. We did not find any support for the *PCLO*-childhood events interaction, but the power of our study was insufficient to exclude this possibility.

## Introduction

Depression results in a high disease burden as well as increased mortality risk due to important impairments in social, emotional and physical functioning. Twin studies found that the genetic contribution to depression is up to 40% [1]. The SNP rs2522833 in the piccolo (*PCLO*) gene has been associated with current depression [2]–[5]. The *PCLO* gene encodes a presynaptic cytomatrix protein called piccolo. Specifically, the rs2522833 polymorphism leads to a serine to alanine substitution in the C2A calcium sensor domain of the protein and may affect protein stability [6], [7]. Monoaminergic modulation of the hypothalamic pituitary adrenal system (involved in stress responses) may be a route through which *PCLO* affects depression vulnerability [4], [8], [9]. The C ‘risk’ allele is more frequent in depressed individuals than in healthy individuals and has been associated with heritable personality traits that predispose to depression in healthy individuals, such as ‘harm avoidance’ [4], as well as with changes in HPA responsiveness after treatment [9]. Other studies, however, were not able to replicate the association with clinical depression [7], or did not find an association with sub-clinical levels of depression [3].

Depression is a complex multifactorial condition and genetic studies so far have yielded mixed results [1]. Cognitive information processing styles associated with depression could be more closely related to genetic factors than the complex and heterogeneous phenotype of major depressive disorder and hence provide another way to study the association between candidate genes and depression [10], [11]. Biased processing of emotional information is considered to be a strong contributor to the development as well as the recurrence of depression, and has also been associated with other forms of psychopathology such as anxiety, eating disorders, and addiction [12]–[17]. Negatively biased *memory*, however, seems particularly robust in depression [18]–[22]. Depressed individuals are inclined to have better memory for negative than positive information, compared to healthy individuals who tend to preferentially recall positive information [18], [19], [23]. Upon remission, sad mood state activates biased processing [24]–[27]. Biased memory in depression is strongest if the information is self-relevant [16], and is generally studied using explicit memory tasks [28]–[30].

Emotional processing biases have been associated with several depression candidate genes, such as the 5-HTTLPR polymorphism in the *SLC6A4* gene, and the *BDNF, COMT* and *NR3C2* genes (e.g. [31]–[36]). In addition to being related to depression, the risk (‘C’) allele in the *PCLO* rs2522833 polymorphism has also been associated with neural correlates of biased processing of emotional faces and memory bias [37]–[39]. Schott and colleagues [27] reported that C allele carriers had lower (non-emotional) memory performance, which was associated with reduced hippocampal activation. Woudstra and colleagues [39] found the *PCLO* polymorphism to affect activation of relevant brain regions (i.e. the anterior cingulate circuit, inferior frontal gyrus, and insula) during emotional memory processing in 89 currently depressed and 29 healthy individuals. However, no direct association between the *PCLO* genotype and emotional memory processing was found.

The complex interplay between genetic susceptibility and stressful life events is considered to be an important mechanism in the development of depression [40]–[42]. Especially stressful childhood events may be of importance [43]. Experiencing negative events in childhood can lead to dysfunctional basic assumptions about the self and the world [44]. These assumptions may induce cognitive biases, as information is processed in accordance with the (dysfunctional) assumptions. Subsequent stressful events may trigger biased processing, such as negatively biased attention and memory, and may contribute to vulnerability for developing symptoms of depression or to relapse (see cognitive models of depression, [44], [45]). This process may be strongest in genetically susceptible individuals. Indeed, studies have found that depression risk allele carriers who experienced stressful life events generally show stronger negative memory bias and/or less positive memory bias [33], [34], [46]. Hence, stressful events may modulate the genetic risk effect on depressotypic information processing.

The fact that the interaction between genetic susceptibility and stressful events has not been examined so far may have contributed to the null-findings regarding the association of *PCLO* and memory bias. Furthermore, most of the participants in the Woudstra and colleagues [39] study were depressed at the time of testing. Participants'depressed mood [16], as well as concentration and memory problems associated with depression probably had a strong effect on emotional memory processing in the Woudstra and colleagues [39] study. This may have overshadowed the small effect of *PCLO* on memory bias. The aim of the current study was to examine the effect of *PCLO* on memory bias as well as the gene-childhood stress interaction in a group of patients remitted from depression and a group of healthy participants. We included a sample of remitted patients because this offers the possibility to study cognitive processes in individuals highly at risk for a (recurrent) depressive episode [47], [48], while minimizing depressive symptoms or concomitants affecting cognitive biases [49]. The analyses were repeated in a sample of healthy individuals to confirm the pattern of results. We hypothesized to find an association between *PCLO* rs2522833 variation and memory bias in remitted depressed patients as well as healthy individuals. We expected this effect to be more pronounced in individuals who experienced stressful childhood events than in individuals who did not.

## Methods and Materials

### Participants

This study is part of a larger study, which aims to link cognitive biases to genetic susceptibility to depression in two independent samples of 360 undergraduate female students of the Radboud University Nijmegen (healthy individuals) and 327 individuals who were remitted from depression (remitted patients). Complete genotype data were available for a total of 351 healthy individuals. Half of the healthy individuals underwent the same sad mood induction procedure as the remitted depressed patients and were therefore selected for the current study. Two of the healthy individuals did not complete the memory task, resulting in a sample of N = 157 for the further analyses. The remitted depressed patients underwent the same sad mood induction. Complete genotype data were available for a total of 320 remitted patients, and complete genotype and memory bias data for a total of 299 remitted patients for further analyses. The remitted patients were recruited at the Department of Psychiatry of the Radboud University Medical Centre and other outpatient psychiatric services in the region. The participants were invited for the study by means of an information letter. Before the onset of the experiment, participants were given the opportunity to ask questions and they were instructed that they could stop at any time, without specification of a reason. Subsequently, they gave written informed consent. Participants were included in the remitted depressed patient group if they met the criteria of the DSM-IV [50] for a previous depressive episode. Exclusion criteria for both groups were: current depressive episode, current or lifetime bipolar disorder, current psychotic symptoms, alcohol or substance abuse within the past 6 months, deafness, blindness, neurological disorder, sensorimotor handicaps and intellectual disability. In order to assess in- and exclusion criteria, trained professionals interviewed eligible participants with the Structured Clinical Interview for the DSM-IV Axis-I disorders (SCID-I) [51]. The SCID-I has been demonstrated to have a good reliability [52], [53]. State depressive symptoms were measured with the Beck Depression Inventory (BDI-II) [54]. Participants received course credit or a gift certificate in return for their participation. Participants were debriefed before they were rewarded for their participation. The study was approved by the Dutch Central Committee on Research involving Human Subjects (CCMO) and we complied with the Code of Ethics of the World Medical Association (Declaration of Helsinki) in the treatment of our participants.

### Stressful childhood events

Stressful childhood events were assessed with an adapted version of the Life Events Questionnaire [55]. This version has previously been used in comparable studies [33], [34], [56]. Participants were asked to indicate whether they had experienced a set of life events before the age 16 of years, after the age of 16, and within the last year. A childhood traumatic events variable was calculated indicating whether or not participants experienced traumatic events (i.e. physical or sexual abuse) involving a family member or a non-family member before the age of 16 years. Because the prevalence of aggression and/or abuse was rather low in the sample of healthy individuals (16% of all participants) and we expect a similar effect of non-traumatic but highly stressful events, the effect of traumatic as well as stressful adverse childhood events was studied in this sample. This variable indicated whether or not participants experienced traumatic events (aggression and/or abuse) involving a family member or a non-family member and/or adverse events (parental loss, divorce of parents and/or prolonged separation of parents) before the age of 16 [33].

### Sad mood induction

Prior to the memory task, participants were presented with a highly emotional sad film segment from the movie “Sophie’s choice” [57]. They were instructed to let the emotionality of the film influence their mood as much as possible and to maintain the sad mood state. Participants rated their mood state using a computerized visual analog scale that ranged from −10 (indicating saddest mood) to 10 (indicating happiest mood) after the mood induction. Remitted patients also rated their mood state before the mood induction.

### Memory bias

This memory task has been previously used and described [35], [58]. Participants were presented with 12 depression-specific negative and 12 positive words in fixed order, with no more than two words of the same valence being presented consecutively. Words were selected from two databases (Dutch translation of the Affective Norms for English Words database; [59, and 60]). Each word was presented for 10 seconds in capital black letters against a white background. To make encoding self-referential, participants were instructed to vividly imagine themselves in a scene with the presented word [61]. After completion of this rating, the next word would appear on the screen. This task was followed by a short paper-and-pencil distraction task (Raven matrices) [62]. After that, participants were instructed to return to the computer for an unannounced free recall of the words from the previous computer task. Participants were instructed to type in all the words they could remember within three minutes. A difference-score representing memory bias was calculated by subtracting the number of correctly recalled negative words from the number of correctly recalled positive words. A negative score represented negative memory bias.

### Genotyping

In healthy individuals, saliva samples were collecting using Oragene kit (DNA Genotek, Inc. Ottawa, Ontario, Canada). In remitted patients, blood was taken by venipuncture and DNA was isolated using standard protocols. Molecular analyses were performed in a CCKL-accredited laboratory at the Department of Human Genetics of the Radboud University Medical Centre. The *PCLO* rs2522833 polymorphism was genotyped using TaqMan analysis [63]. Genotyping failed for two remitted patients and nine healthy individuals, resulting in a call rate of 99.4% for remitted patients and 97.5% for healthy individuals, respectively. The *PCLO* allele frequency distribution in healthy individuals and remitted patients is presented in Table 1. Testing for Hardy–Weinberg equilibrium did not show deviation from the expected distribution of genotypes in healthy individuals, N = 351, χ^2^ = .10, df = 1, *p* = .75, nor in remitted patients, N = 320, χ^2^ = .03, df = 1, *p* = .85. Genotype groups were created in line with Woudstra and colleagues [39] and the association with depression: ‘C’ risk allele carriers (homozygous and heterozygous for C allele) versus ‘A’ non-risk allele carriers (homozygous for A allele).

**Table 1:** The PCLO allele frequency distribution presented by number of participants and percentage of participants in remitted patients and healthy individuals.

	Remitted patients (N = 320)	Healthy individuals (N = 351)
Genotype	N (%)	N (%)
C/C	57 (18%)	67 (19%)
C/A	158 (49%)	176 (50%)
A/A	105 (33%)	108 (31%)

### Statistical analyses

The effects of *PCLO* genotype (‘C’ risk allele carriers, ‘A’ non-risk allele homozygotes), childhood trauma/adversity (no, yes) and their interaction on memory bias in remitted patients and healthy individuals were examined using analysis of co-variance (ANCOVA). In line with the Woudstra and colleagues [39] study, we also examined the two samples together. Because of their known effect on cognitive functioning and emotional processing, age and sex were included as covariates in the analyses.

## Results

### Sample descriptive and mood induction

Descriptives of the two independent samples are presented in Table 2. Pre mood induction mood ratings of 14 remitted patients were missing due to a programming error. The mood induction was successful in remitted patients as mood state decreased significantly from pre (M = 3.47 S.D.3.62) to post (M = .27 S.D. = 4.52), F(1,284) = 236.91, *p*<.001, Cohen's *f* = .91. The effect in healthy participants could not be evaluated because we did not have a baseline mood measure.

**Table 2:** Demographic and childhood events variables of the two independent samples. Childhood trauma refers to physical or sexual abuse before the age of 16 years and childhood adversity refers to childhood trauma and parental loss, divorce of parents and/or prolonged separation of parents before the age of 16. SD refers to standard deviation.

	Remitted patients (N = 299)	Healthy individuals (N = 157)
Variable	Mean (SD)/%	Mean (SD)/%
Age (years)	47.6 (11.9)	21.1 (3.7)
Sex (% female)	65%	100%
Childhood trauma (yes/no)	38%	15%
Childhood adversity (yes/no)	55%	48%

### *PCLO* genotype main effect

Neither in remitted patients, *F*(1,293)  = 1.89, *p* = .170, *f* = .08, nor in healthy individuals, *F*(1,152)  = .83, *p* = .363, *f* = .07. *PCLO* a main effect of *PCLO* genotype on memory bias was found. The analysis of the whole two samples together did not yield an effect on memory bias either , *F*(1,450)  = 2.15, *p* = .144, *f* = .06.

### *PCLO* genotype x stressful childhood events interaction

The mean memory bias difference scores (# correctly recalled positive words - # correctly recalled negative words) are presented in Table 3. In remitted patients, genotype did not significantly interact with childhood trauma on memory bias, *F*(1,293)  = 1.18, *p* = .184, *f* = .08. The results are similar when examining the interaction between genotype and childhood adversity (as in healthy individuals) instead of childhood trauma in remitted patients, *F*(1,293)  = .14, *p* = .713, *f* = .00. The interaction between genotype and childhood adversity was not significant in healthy individuals either, *F*(1,152)  = 3.09, *p* = .081, *f* = .14.

**Table 3:** Mean memory bias difference scores (# correctly recalled positive words - # correctly recalled negative words) and standard deviations (SD) for the PCLO genotype (risk allele ‘C’ carriers vs. non-risk allele ‘A’-homozygotes) x childhood trauma interaction in remitted depressed individuals, and for the PCLO genotype x childhood adversity interaction in healthy individuals.

Genotype	Childhood trauma	Memory bias Mean (SD)
Remitted patients		
Risk allele carriers	No	1.24 (1.79)
	Yes	1.06 (2.01)
Non-risk allele carriers	No	1.24 (1.64)
	Yes	1.68 (1.99)
**Healthy individuals**	**Childhood adversity**	**Memory bias Mean (SD)**
Risk allele carriers	No	.88 (1.69)
	Yes	.94 (1.99)
Non-risk allele carriers	No	1.74 (1.97)
	Yes	.68 (2.18)

A higher score represents more positive relative to negative memory bias.

Restricting the analyses to female patients with remitted depression (N = 193) did not make any difference in terms of *PCLO* main effect, *F*(1,188)  = .93, *p* = .336, *f* = .07, or interaction with childhood trauma, *F*(1,188)  = .42, *p* = .520, *f* = .04.

No significant effect was found when examining the genotype by childhood adversity interaction in the two samples together, *F*(1,450)  = .57, *p* = .451, *f* = .03. To check whether our non-significant results were due to a lack of statistical power, we conducted post hoc power analyses using Quanto power calculator (http://hydra.usc.edu/gxe/). The power analyses revealed that in order for a *PCLO* main effect of this size to be detected (80% chance) as significant at the 5% level, a sample of 134 participants would be required, and 2153 participants for an interaction effect.

## Discussion

Previous studies have found an association between the *PCLO* rs2522833 polymorphism and neural correlates of biased processing in healthy and currently depressed individuals, but no direct effect of the *PCLO* genotype on memory bias [38], [39]. Memory bias has been associated with other depression candidate genes, specifically in individuals who experienced stressful life events (e.g. [33], [34]). Therefore, we hypothesized that the absence of the association between the *PCLO* genotype and memory bias in previous studies might have been due to genetically driven individual differences in reactivity to environmental stress [41], as well as to current depressive symptoms. The association between the *PCLO* rs2522833 polymorphism, memory bias and the modulating effect of stressful childhood events was examined in a sample of remitted depressed patients and a healthy sample. We found no evidence for an association between the *PCLO* rs2522833 polymorphism and memory bias, nor for an interaction effect of the polymorphism with stressful childhood events. Our findings add to the current knowledge and suggest that the absence of an association between *PCLO* and memory bias may not be explained by current depressive symptoms, or by a gene-childhood stress interaction. However, the current study did not have sufficient power to detect the *PCLO*-childhood stress interaction and may have resulted in false negative findings. Therefore, we cannot rule out the possibility that the interaction effect exists, but was not detected due to a lack of power. A larger sample is required to confirm these results. Taken together, results from our study and the recent study by Woudstra and colleagues [39] indicate that verbal emotional memory bias may not be strongly associated with the *PCLO* rs2522833 polymorphism - unlike other genes that have been associated with both memory bias and depression (e.g. *BDNF*, *NR3C2*) - and may be implicated in other routes towards the susceptibility for depression.

It has been proposed that the *PCLO* rs2522833 polymorphism may be one of the genetic variants that increase the risk for depression by affecting the stress regulation capacity of the brain, possibly by altering monoaminergic neurotransmission, especially serotonin [39]. The imaging studies offer support for this [38], [39]. Variation in the *PCLO* rs2522833 polymorphism was associated with amygdala activation during emotion processing, as well as lower striatum activation during emotional memory processing in depressed individuals. Altered activation in these neuronal circuits is related to depression and emotional information processes [64]. Based on the endophenotype model [10], the path from gene to brain activation is shorter and the link stronger, compared to the path from gene to cognitive measures, such as memory bias. Hence, the effects of *PCLO* may be too subtle to show up on a cognitive measure, while effects can be found on neural correlates of biased processing. We included, however, a larger sample than the previous study to examine the association of *PCLO* with memory bias [39] and we had sufficient power to detect a main effect of the *PCLO* polymorphism. The null-findings may therefore imply that the *PCLO* rs2522833 polymorphism is not related to emotional information processing that is associated with the susceptibility for depression.

Previous studies found an association between depression and the *PCLO* rs2522833 polymorphism when studying current major depressive disorder, but not for sub-clinical levels of depression [3], [7], [65]. To illustrate, in the study by Hek and colleagues [3], evidence for the association with depression was found in a well-diagnosed clinical subsample, but not in a sample of individuals with minor depression, self-reported depression, depression diagnosed by a general practitioner, or individuals using antidepressant medication. Sullivan and colleagues [7] also only found an association between *PCLO* and depression in a homogeneous clinically depressed subsample. Patients included in our study fulfilled the clinical criteria of one or more depressive episodes in the past. Our null-findings can therefore not be attributed to the selection of a too heterogeneous sample with subclinical levels of depression. However, it is important to note that we studied *PCLO* in relation to information processing styles that are associated with depression and the susceptibility for depression only, and not the association between *PCLO* and depressive disorder, as in the studies presented above.

Our results should be viewed in the context of some strengths and limitations. Strengths of our study are that we used two samples and that studying gene-bias associations in an affected sample is rather novel. Another strength of the study is that we avoided current symptoms overruling subtle cognitive effects by testing groups that are currently not depressed. A limitation is the difference in sex distribution between the two samples, as SNPs within the *PCLO* gene have been associated with depression in females and not in males [66]. The fact that only female healthy individuals were included, limits the generalizability of these results. Another limitation may be the measurement method of the childhood events, as negative bias may have affected the participants' recall. The Life Events Questionnaire assesses the occurrence of several factual events during pre-determined periods of life. Because such specific events were assessed and generally adults' recall of specific childhood events is fairly accurate [67], it is in our opinion unlikely that recall had been distorted.

In conclusion, the presence of the *PCLO* risk allele may increase vulnerability for depression, but we found no indication for an association with memory bias. Memory bias may not be strongly associated with the *PCLO* rs2522833 polymorphism, unlike other genes that have been associated with depression (e.g. *BDNF, NR3C2*). Due to the insufficient power to detect a *PLCO*-childhood trauma interaction effect, we however cannot rule out that *PLCO* is associated with memory bias in individuals with stressful childhood events.
